# Parosmia, Dysgeusia, and Tongue Features Changes in a Patient with Post-Acute COVID-19 Syndrome

**DOI:** 10.1155/2021/3788727

**Published:** 2021-08-26

**Authors:** Aliaa Abdelmoniem Bedeir Eita

**Affiliations:** Faculty of Dentistry, Oral Medicine, Periodontology, Diagnosis and Radiology Department, Alexandria University, Alexandria, Egypt

## Abstract

Coronavirus disease 2019 (COVID-19) is the pandemic of major global concern that has been causing several tragic events ever since December 2019. Infection with COVID-19 varies from being asymptomatic or mild with general unwellness to severe with difficulty in breathing and even mortality. Recently, several reports signified the persistence of symptoms or the development of new ones for weeks or months after the virus has gone, the so-called “post-acute COVID-19 syndrome.” This article presents a 31-year-old female with post-acute COVID-19 syndrome showing parosmia, dysgeusia, and tongue feature changes as simultaneous newly developed manifestations after the viral clearance. Follow-up revealed complete remission of manifestations after one month. Post-acute COVID-19 syndrome can show oral manifestations. Despite the lack of evidence regarding the etiology, risk factors, and consequences of post-COVID-19 syndrome, it is important to monitor COVID-19 survivors to avoid complications during their recovery period.

## 1. Introduction

Coronavirus disease 2019 is caused by severe acute respiratory syndrome coronavirus 2 (SARS-CoV-2), an enveloped RNA virus and a member of the *Coronaviridae* family [1]. Infection with SARS-CoV-2 elicits an array of clinical manifestations that mostly include fatigue, fever, dry cough, and others ranging from mild to severe [2]. Several oral manifestations have also been reported in patients with acute COVID-19 infection. Dysgeusia (altered taste sensation) is considered an early recognized symptom which is believed to help in the early diagnosis of the disease. Other oral findings include ulcers, vesiculo-bullous eruptions, petechiae, erythema, tongue coat changes (white, yellow, and/or greasy tongue), and more [3–5].

Post-acute COVID-19 syndrome, also called “long COVID,” “COVID long haul syndrome,” and “post-COVID syndrome” (PCS), is known as the persistence of symptoms or the development of complications for more than three to four weeks after the onset of acute viral symptoms and where SARS-CoV-2 is not isolated [6, 7]. Fatigue, insomnia, compromised memory and concentration, palpitations, gastrointestinal manifestations, parosmia, and dysgeusia are all common symptoms of long COVID-19 [7]. Serious manifestations as acute renal injury, diabetic ketoacidosis, and multisystem involvement were also reported [6].

The aim of this report is to contribute to the current literature by presenting a case of the newly described entity post-acute COVID-19 syndrome showing parosmia (altered smell sensation), dysgeusia, and greasy white coated tongue with prominent papillae as simultaneous newly developed manifestations for four weeks after polymerase chain reaction (PCR) negativity. To the best of our knowledge, this is the first case report to document tongue manifestations in PCS.

## 2. Case Report

A 31-year-old female patient presented to the oral medicine clinic complaining of dysgeusia; she lost the ability to distinguish food with the retention of sweet and salty sensations only. Moreover, she was concerned about an unpleasant greasy white tongue and a distorted sense of olfaction where everything smelled like chemicals (parosmia). The patient reported the onset of these symptoms two days earlier with significant worsening. The condition was persistent and affected her quality of life, the thing that made her seek professional assistance.

### 2.1. History, Clinical Examination, and Diagnosis

Past medical history revealed that the patient is atopic; she reported occasional administration of antihistamines (loratadine 10 mg once daily) whenever hypersensitivity reactions are experienced. The patient was also suffering from episodes of irritable bowel syndrome for ten years. She is used to administering simethicone (125 mg 2-3 times daily) during exacerbations to relief bloating. Dental and family histories were negative.

Fourteen days earlier, the patient was COVID-19 positive. She suffered from sore throat, persistent low-grade fever (38°C), nasal congestion and itching, conjunctivitis, nausea, abdominal pain, diarrhea, fatigue, and severe joint pain. Chest computed tomography and all the relevant laboratory investigations were normal. The patient was scheduled on a treatment protocol for COVID-19 in accordance to her current medical condition following the guidelines set by the Egyptian Ministry of Health and Population [8] and under the supervision of a physician. Therapy included azithromycin 500 mg/day, paracetamol 500 mg 2 tablets ×3 times daily, zinc (50 mg), and vitamin C (1000 mg) supplements once daily. Throughout the treatment phase, symptom improvement was noted. After 12 days of infection, a nasopharyngeal swab was collected, and real-time PCR for COVID-19 detection was negative. At that time, all the reported COVID-19 symptoms disappeared, and treatment was discontinued.

**Figure 1:**
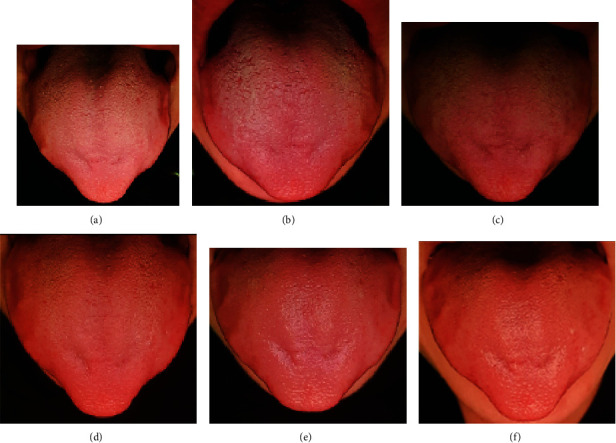
(a–f) Tongue feature changes along four weeks of follow-up. Week one (a, b): (a) visit 1 showing white coated tongue with prominent papillae; (b) visit 2 showing increased tongue coat thickness over the dorsal lateral aspects with prominent papillae. Week two (c, d): (c) visit 3 showing case stability from visit 2; (d) visit 4 showing improvement with restoration of the normal tongue coating and papillary features. Weeks three and four (e, f) showing further improvement than the previous weeks with normal tongue coating and papillae.

Extraoral examination was normal. Intraoral examination revealed an unscrapable white coat over the dorsum of the tongue with prominent papillae. Bilateral indentation marks from reported continuous tongue sucking were also noted (Figure 1(a)). Tongue sucking was an established oral habit ever since childhood; underlying medical conditions and psychological impairment were ruled out.

Based on history and clinical examination, the patient was diagnosed with post-acute COVID-19 syndrome.

### 2.2. Treatment and Follow-Up

The patient was reassured, and a comprehensive explanation about her condition was performed. She was instructed about the appropriate oral hygiene measures, and prophylactic medications were prescribed to prevent superimposed infections (0.12% chlorhexidine mouth rinse and nystatin oral drops 100.000 I.U/ml 2-3 times daily). Daily vitamin C (1000 mg) and zinc (50 mg) supplements were also prescribed to enhance recovery.

Follow-up appointments were scheduled at short intervals (one to two visits per week throughout four weeks) to monitor manifestation improvement (Figures 1(a)–1(f)). Phone calls were arranged every other day from the start of treatment to ensure patient's compliance with the scheduled protocol. A second PCR test for COVID-19 detection was performed by the end of week one follow-up and confirmed the previous negative result.

**Figure 2:**
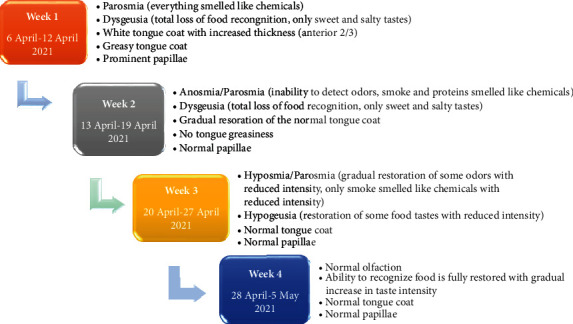
Timeline of the patient's symptoms throughout four weeks of post-acute COVID-19 syndrome.

After one month of treatment (4 weeks from viral clearance), the tongue features were restored to normal, and the patient reported complete resolution of parosmia and dysgeusia. No adverse side effects were noted. A detailed documentation of the changes in all manifestations of PCS is shown as a timeline in Figure 2. The patient was followed up after another month from PCS recovery (8 weeks from viral clearance) and showed no complication or symptom recurrence.

## 3. Discussion

Coronavirus disease 2019 outbreak has been gaining the world's attention to date. Several studies have discussed the nature and manifestations of COVID-19. However, scientists cannot predict its course and sequelae in patients during and after the viral infection [1].

There is a growing body of evidence about the symptoms and risk factors of PCS. In the present case, the patient's sex and some of the clinical features at the viral stage as persistent fever and infection duration are coincident with the emerging literature. Mahmud et al. [9] in their cohort study reported fatigue as the most prevalent symptom. Moreover, they reported female sex, lethargy, respiratory distress, and long infection durations (14 days from PCR diagnosis) as risk factors for PCS. In the same study, patients with fever were found to be more susceptible to develop PCS.

Olfactory dysfunction and dysgeusia have been notably discussed in the context of SARS-CoV-2 infection. The pathogenesis of anosmia (loss of smell sensation) in COVID-19 can be explained by several mechanisms like nasal airway obstruction, olfactory center and sensory neural damage, olfactory epithelium, and supporting cell dysfunction. On the other hand, it was found that salivary glands and the oral mucosa are vulnerable to being attacked by SARS-CoV-2 through angiotensin converting enzyme 2 receptors (ACE2) leading to dysgeusia. Other mechanisms for dysgeusia have been proposed as zinc deficiency, cranial nerve dysfunction, and others [10].

In general, chemosensory dysfunction (olfactory and gustatory) was found to be linked to stress, anxiety, and depression. Depression was found to be associated with high levels of glucocorticoids and inflammatory cytokines which can result in compromised olfactory neural proliferation [11]. Moreover, a study by Al'Absi et al. [12] reported that stress-related changes in the adrenocortical activity were linked to altered taste perception. In COVID-19 patients, Speth et al. [13] revealed a positive and specific association between the severity of taste and smell loss and depressed mood and anxiety from viral infection. The authors raised attention to direct viral pathogenesis as a possible cause of such an association.

Different tongue coat changes were reported in COVID-19 patients. Pang et al. [4] found that light red tongue with white coating is commonly seen in mild to moderate SARS-CoV-2 infection. Furthermore, they reported greasy coating as a significant characteristic in all patients. White coated tongue is usually a normal physiologic change. Regardless, white and red oral mucosal patches or plaques seen in COVID-19 patients could be attributed to superimposed fungal infection from long-term treatments or difficulties in complying with the meticulous oral hygiene measures from health deterioration [3]. Oxidative stress and dysbiosis have been linked to both COVID-19 infection and greasy tongue coating [14, 15]. It was suggested that the resultant cellular damage from oxidant-antioxidant imbalance as well as dysbiosis could be possible causes for greasy tongue coating in COVID-19 patients [3].

To date, the exact pathophysiology of PCS is not well established. Nonetheless, the implication of physiologic viral changes, host immune, and inflammatory response to the invading pathogen and critical illness have all been proposed as possible mechanisms of PCS [6]. The present patient suffered from persistent nasal itching and fever throughout the acute viral phase. Besides, she was tired and unable to follow the appropriate measures of care. The reported manifestations of PCS in this article could be the sequelae of such compromisations, the result of the patient's body response to face COVID-19, or a composite of all proposed mechanisms.

Due to the increasing sequelae of COVID-19 in the postviral stages on a multiorgan scale, studies highlighted the importance of monitoring COVID-19 patients for longer durations after the viral clearance. This would help understand more about this pandemic through rigorous investigation, management, and documentation of the newly evolving symptoms. The provided care should involve multidisciplinary cooperation in both inpatient and outpatient settings in accordance to the prevailing symptoms and their severity [6].

One possible limitation of the present case is the lack of reagents and materials used for olfactory and taste function tests. Chemosensory function testing would have provided more details about the changes of parosmia and dysgeusia throughout the treatment and follow-up durations.

## 4. Conclusion

Coronavirus disease 2019 is an infectious disease with heterogeneous literature and international health challenges. New symptoms can develop post-COVID-19 clearance. Parosmia, dysgeusia, and white tongue coating with greasiness can be symptoms of post-acute COVID-19 syndrome. Meticulous patient care through long-term follow-up is crucial to avoid complications during convalescence.

## Data Availability

All data supporting the conclusions of this report is available in the manuscript.

## Additional Points

*Patient Perspective*. The patient reported overall satisfaction at the end of follow-up. The odor of chemicals and the greasy tongue feeling have completely disappeared. Also, she was able to distinguish between all food types.
